# High-Dimensional Analysis Reveals Distinct Endotypes in Patients With Idiopathic Inflammatory Myopathies

**DOI:** 10.3389/fimmu.2022.756018

**Published:** 2022-02-21

**Authors:** Erin M. Wilfong, Todd Bartkowiak, Katherine N. Vowell, Camille S. Westlake, Jonathan M. Irish, Peggy L. Kendall, Leslie J. Crofford, Rachel H. Bonami

**Affiliations:** ^1^ Division of Rheumatology and Immunology, Vanderbilt University Medical Center, Nashville, TN, United States; ^2^ Division of Allergy, Pulmonary, and Critical Care Medicine, Vanderbilt University Medical Center, Nashville, TN, United States; ^3^ Vanderbilt Institute for Infection, Immunology, and Inflammation, Vanderbilt University Medical Center, Nashville, TN, United States; ^4^ Human Immunology Discovery Initiative and Vanderbilt Center for Immunobiology, Vanderbilt University Medical Center, Nashville, TN, United States; ^5^ Department of Cell and Developmental Biology, Vanderbilt University, Nashville, TN, United States; ^6^ Vanderbilt-Ingram Cancer Center, Vanderbilt University Medical Center, Nashville, TN, United States; ^7^ Deparment of Pathology, Microbiology, and Immunology, Vanderbilt University Medical Center, Nashville, TN, United States; ^8^ Department of Medicine, Division of Allergy and Immunology, Washington University School of Medicine St. Louis, MO, United States

**Keywords:** idiopathic inflammatory myopathies (IIM), dermatomyositis (DM), polymyositis (PM), mass cytometry (CyTOF), immunophenotype

## Abstract

The idiopathic inflammatory myopathies (IIM) are a rare clinically heterogeneous group of conditions affecting the skin, muscle, joint, and lung in various combinations. While myositis specific autoantibodies are well described, we postulate that broader immune endotypes exist in IIM spanning B cell, T cell, and monocyte compartments. This study aims to identify immune endotypes through detailed immunophenotyping of peripheral blood mononuclear cells (PBMCs) in IIM patients compared to healthy controls. We collected PBMCs from 17 patients with a clinical diagnosis of inflammatory myositis and characterized the B, T, and myeloid cell subsets using mass cytometry by time of flight (CyTOF). Data were analyzed using a combination of the dimensionality reduction algorithm t-distributed stochastic neighbor embedding (t-SNE), cluster identification, characterization, and regression (CITRUS), and marker enrichment modeling (MEM); supervised biaxial gating validated populations identified by these methods to be differentially abundant between groups. Using these approaches, we identified shared immunologic features across all IIM patients, despite different clinical features, as well as two distinct immune endotypes. All IIM patients had decreased surface expression of RP105/CD180 on B cells and a reduction in circulating CD3+CXCR3+ subsets relative to healthy controls. One IIM endotype featured CXCR4 upregulation across all cellular compartments. The second endotype was hallmarked by an increased frequency of CD19+CD21loCD11c+ and CD3+CD4+PD1+ subsets. The experimental and analytical methods we describe here are broadly applicable to studying other immune-mediated diseases (e.g., autoimmunity, immunodeficiency) or protective immune responses (e.g., infection, vaccination).

## Introduction

The idiopathic inflammatory myopathies (IIM) are a family of autoimmune diseases afflicting 2.4 to 33.8 per 100,000 individuals. IIM are associated with significant morbidity and mortality (1). IIM patients have a three-fold increased risk of death (2), and 14% cannot dress independently (3). Clinically, IIM are quite heterogenous and causes varying degrees of skin rash, proximal muscle weakness, esophageal dysmotility, and interstitial lung disease (ILD). The presence of different autoantibodies is associated with specific clinical phenotypes (4–15). For example, Mi-2 positive patients frequently manifest with classical rashes, mild to moderate muscle involvement, and rarely have ILD (15), whereas MDA5 positive patients have cutaneous ulcerations, arthritis, and progressive lung disease that can rapidly lead to death (14). Immune subsets interact and influence each other (16–23). B cells, T cells, dendritic cells, and monocytes have been independently characterized in IIM patients (24–29), but all compartments have not been immunophenotyped in tandem from the same patient. Recent technical and analytical advances create an opportunity to probe relational changes in all of these subsets simultaneously to build a more complete picture of immune dysfunction in IIM.

Cryopreserved PBMC samples enable longitudinal studies of the same donor as one batch. Aliquots of the same sample can also be run in different assays. Several groups have shown that cryopreserved lymphocytes approximate fresh *ex vivo* results (30–33). To improve our understanding of IIM immunophenotypes across all PBMC compartments, we performed detailed immunophenotyping using mass cytometry by time of flight (CyTOF) to analyze cryopreserved PBMC samples isolated from a cohort of IIM patients with active disease compared to healthy controls. A challenge in analyzing mass cytometric data is the number of parameters analyzed simultaneously. While canonical immune subsets (34) and previously described autoimmune-prone subsets (35–40) can be analyzed using traditional biaxial gating, dimensionality reduction tools, such as t-SNE and UMAP, facilitate discovery of non-canonical immune subsets that may otherwise be missed by manual biaxial gating approaches that require *a priori* knowledge to identify subsets. Dimensionality reduction tools convert plots of cells in multi-dimensional space into a two-dimensional map for manual review by scientists (41). In contrast, clustering algorithms, such as CITRUS (42) and FlowSOM (43), use automated approaches to detect and define cell populations for downstream analyses, including manual biaxial gating. While FlowSOM stands out compared to other unsupervised clustering tools due to its fast and accurate grouping of cells by phenotype, FlowSOM does not analyze external categories as part of finding cell clusters (44). In contrast, CITRUS is a statistically robust tool for supervised analysis, meaning that it allows the user to guide the analysis using an external categorization of comparison groups and incorporates key statistical concepts like false discovery rate. Additional algorithms, such as marker enrichment modeling (45), can further characterize these clusters and facilitate the development of biaxial gating schemes for cluster validation. We therefore elected to use t-SNE, as well as CITRUS and MEM to identify immune perturbations that distinguished groups and manual biaxial gating to validate proportional differences in these putative populations between groups.

Previously, CD21lo/neg (46) and CD180lo (37) B cell subsets were found to be increased in IIM. We hypothesized that machine learning would identify additional atypical B cell populations. Therefore, in addition to measuring perturbation of canonical PBMC (34) and autoimmune prone B cell subsets (35–37, 39, 40) in IIM patients *via* biaxial gating, we employed t-SNE to visualize the B cell immunophenotypes in IIM and identify additional atypical populations. We then grouped patients by the presence of atypical B cell subsets and exploited the statistical robustness of CITRUS to identify other PBMC subsets associated with the t-SNE identified B cell subsets, which would support the hypotheses that immune endotypes exist within IIM. Such immune endotypes may have important clinical correlates such as disease severity, end-organ involvement, or treatment response.

## Materials and Methods

### Patients

Institutional Review Board approval was obtained (VUMC IRB 141415). Patients clinically diagnosed with IIM were enrolled into the Myositis and Scleroderma Treatment Initiative Center (MYSTIC) Cohort in either the outpatient clinic or inpatient setting at Vanderbilt University Medical Center between 9/17/2017 and 9/30/2018. Individuals enrolling as healthy controls completed a health questionnaire to verify a negative review of systems and no personal or family history of autoimmunity in a first degree relative. We performed clinical phenotyping by chart abstraction to estimate the date of symptom onset and collected serologic data, including anti-nuclear antibodies (ANA), rheumatoid factor (RF), cyclic citrullinated peptide (CCP), and an extended myositis panel obtained through ARUP (Salt Lake City, UT), which included Jo-1, PL-7, PL-12, EJ, OJ, Ro52, Ro60, Ku, MDA5, Mi-2, NXP-2, P155/140, Pm/Scl 100, SAE-1/SUMO, SRP, Tif-1γ, U1RNP, and U3RNP. We defined patients as having ILD if a radiologist determined that fibrosis was present on a CT scan. If the treating clinician escalated immunosuppression, the patient was defined as having active disease. Clinical data are reported as the mean ± standard deviation unless otherwise indicated. We isolated peripheral blood mononuclear cells (PBMCs) from blood collected in sodium heparin CPT tubes (BD Biosciences, San Jose CA) per manufacturer’s directions and cryopreserved for future study.

### Mass Cytometry

Seventeen IIM patients with active disease and eighteen healthy controls were included for CyTOF analysis. For CyTOF acquisition, we thawed 3-5 million PBMCs per individual, viability stained with cisplatin, and stained for surface and intracellular markers (see **Supplemental Methods** and **Tables S1, S2**). Data were acquired using a CyTOF Helios 3.0 (Fluidigm Sciences, Sunnyvale, CA) and CyTOF software (version 6.7.1014) at the Vanderbilt University Medical Center Mass Cytometry Center of Excellence. Dual count calibration and noise reduction were applied prior to acquisition; 100,000-400,000 events were collected per sample.

### Flow Cytometry

We selected six representative IIM patients and six healthy controls for flow cytometric studies of CD180 expression, for which 4-5 million cells per individual were thawed and stained (see **Supplemental Methods** and **Table S3**). We measured intracellular and extracellular CD180 levels simultaneously on the same day. All data were acquired on a BD LSRII Fortessa instrument.

### Data Analysis

Mass cytometry FCS files underwent Fluidigm bead normalization and analysis using Cytobank software per established methods (47). Dimensionality reduction (t-SNE (41) and UMAP (48)), clustering (CITRUS (42)), or supervised (traditional biaxial gating) analyses were conducted using CytoBank (Santa Clara, CA). Marker enrichment modeling (MEM) labels (45) aided in determining biaxial gating schemes for CITRUS-identified populations. Details of mass cytometric data analysis are included in the **Supplemental Methods** and **Tables S4, S5**. Fluorescence cytometry data was analyzed using FlowJo version 9.9.6.

### Statistical Methods

Population statistics were exported from CytoBank or FlowJo as appropriate and analyzed with Prism software (GraphPad, La Jolla, CA) to calculate descriptive statistics. Data are expressed as mean ± standard deviation unless otherwise indicated. If multiple groups were compared, we performed a Kruskal-Wallis ANOVA and, if p<0.05, *post-hoc* Mann-Whitney U-tests were performed. For comparison of two continuous variables, we utilized Mann-Whitney U-tests. For comparison of two dichotomous variables, a Fisher’s exact test was performed. Spearman’s correlation coefficients were utilized to identify the presence of a statistical correlation between populations.

## Results

### Patient Characteristics

We studied 17 patients clinically diagnosed with IIM and 18 healthy controls. Basic demographics and clinical information are shown in **Table 1**. Detailed clinical phenotyping is shown in **Supplemental Table S6**. IIM patients were slightly older than healthy controls (56.8 ± 12.0 v. 46.4 ± 12.0 years, p=0.01). Seven patients were receiving corticosteroids at the time of enrollment; one patient was taking methotrexate at the time of enrollment with active skin, lung, and joint involvement. Eleven patients met the 2017 classification criteria for probable or definite IIM. Those not meeting classification criteria all had interstitial lung disease and myositis-specific autoantibodies.

**Table 1:** Patient demographics.

	IIM Patients	Healthy Controls
	(n = 17)	(n = 18)
Average Age	56.8 ± 11.7	46.4 ± 11.7
Female Gender	8 (44.4%)	14 (82%)
Race		
Caucasian	13 (76.4%)	16 (88.8%)
African American	4 (23.5%)	1 (5.6%)
Other	0 (0%)	1 (5.6%)
Average Disease Duration in Months (median, IQR)	10.8 (8.4,15.6)	
Clinical Categorization		
Dermatomyositis	6 (35.3%)	
Polymyositis	4 (23.5%)	
Anti-synthetase syndrome	7 (41.2%)	
Interstitial Lung Disease	14 (82.3%)	
Average % predicted FVC (n=11)	56.9 ± 11.9%	
Average % predicted DLCO (n=9)	49.1 ± 15.1%	
Supplemental oxygen use at enrollment	6 (42.9%)	
History of elevated creatinine kinase	9 (52.9%)	
Serologic Status		
+ANA	8 (47.0%)	
+Rheumatoid factor (n=14)	5 (35.6%)	
+Ro52	6 (35.3%)	
+Myositis specific or associated autoantibody	16 (94.1%)	
Probable or definite idiopathic inflammatory myositis according to 2017 classification criteria (49)	11 (64.7%)	

Data reported as mean ± standard deviation unless otherwise noted.

FVC, forced vital capacity; DLCO, diffusing capacity of the lung for carbon monoxide; ANA, anti-nuclear antibodies.

### Lymphoid and Myeloid Subsets Are Altered in IIM Patients Compared to Healthy Controls

**Figure 1:**
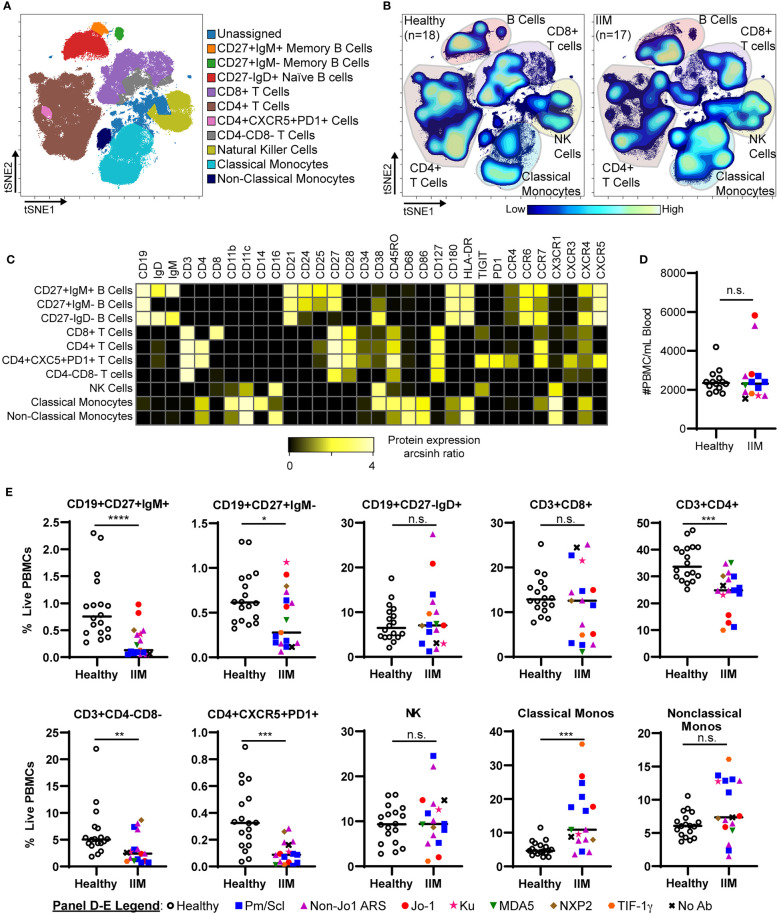
IIM patients show differences in peripheral blood immune subsets from healthy controls. **(A)** Live PBMCs from healthy controls (n=18) and IIM patients (n=17) were visualized using t-SNE and manually assigned to traditional immune cell populations (indicated by color). **(B)** Density contour maps of concatenated healthy controls versus IIM patients. **(C)** Expression heatmaps of representative markers for t-SNE islands displaying the arcsinh ratio by table’s minimum. **(D, E)** Clinical seropositivity for the indicated autoantibodies is shown by legend symbols at the bottom of Panel **(E)**. Individual donors are plotted. **(D)** Absolute number of circulating PBMCs for healthy controls (n=14) and IIM patients (n=14) for whom count data were available. **(E)** Comparison of major circulating PBMC subsets between healthy controls and IIM patients using t-SNE visualization and manual gating from panel **(A)**. Statistical comparisons were performed using Mann-Whitney U tests. *p < 0.05, **p < 0.01, ***p < 0.001, ****p < 0.0001, not significant (n.s.).

We evaluated common myeloid, T, and B cell subsets based on standardized immunophenotypic markers (34) using traditional biaxial gating (**Supplemental Figure S1**). Regions for each subset are pseudocolored on a t-SNE plot including all donors concatenated (**Figure 1A**) or divided into concatenated healthy control or IIM t-SNE plots, in which major subset regions are indicated by shading (**Figure 1B**). **Figure 1C** shows a heatmap of all markers for each population to ensure all populations were correctly identified. **Table 2** shows the average, standard deviation, and p value comparing all populations between IIM and healthy controls based on manually-defined populations on t-SNE plots. Complete blood count with differential was available for 14 IIM patients and 14 healthy controls. There was no difference between the number of circulating PBMCs between IIM patients and healthy controls (**Figure 1D**). There was no difference in the percent of live cells for healthy controls compared to IIM patients (92.2 ± 5.1% v. 94.2 ± 2.7%, p=0.27). As shown in **Figure 1E**, IIM patients had a decreased frequency of class-switched (CD19+CD27+IgM-) and non-class-switched (CD19+CD27+IgM+) memory B cells compared to healthy controls; there was no difference in the frequency of naïve (CD19+CD27-IgD+) or total B cells. While there was no statistically significant difference in CD8+ T cells, there was a decrease in CD4+, CD4-CD8- and CD4+CXCR5+PD1+ T cells. Classical monocytes (CD14+CD16-CD19-CD3-) were increased in IIM patients compared to healthy controls, but there was no difference in the frequency of natural killer cells (CD16+CD19-CD3-CD14-) or non-classical monocytes (CD14+CD16+CD19-CD3-).

**Table 2:** Peripheral blood mononuclear cell (PBMC) subsets and median mass intensities (MMI) in healthy controls and idiopathic inflammatory myopathy (IIM) patients.

Population	Healthy	IIM	P value
	(n = 18)	(n = 17)	
Total number of circulating PBMCs (cells/mL)	2443 ± 614	2649 ± 1301	0.72
CD19+			
Total CD19+^†^	10.3 ± 4.3%	10.8 ± 7.8	0.61
CD19+CD27- (naïve) ^‡^	7.5 ± 4.0%	8.6 ± 6.9%	0.96
CD19+CD27+IgM+ (non-class switched memory) ^‡^	0.9 ± 0.6%	0.3 ± 0.3%	<0.0001
CD19+CD27+IgM- (class switched memory) ^‡^	0.7 ± 0.3%	0.4 ± 0.3%	0.03
CD19+CD24hiCD38hi^‡^	3.8 ± 1.7%	4.9 ± 5.1%	0.66
CD19+CD21lo^‡^	5.8 ± 2.2%	11.7 ± 7.2%	0.001
CD19+CD27-IgD- (DN B cells) ^‡^	7.4 ± 2.9%	12.7 ± 5.8%	0.002
CD19+CD27-IgD+IgM- (BND cells) ^‡^	2.7 ± 1.0%	2.6 ± 1.7%	0.33
CD19+ CD180 MMI	60.9 ± 20.8	39.8 ± 16.0	0.002
CD19+CD27- CD180 MMI	58.3 ± 22.5	38.5 ± 16.3	0.009
CD19+CD27+IgM+ CD180 MMI	80.9 ± 19.1	58.5 ± 15.2	0.0005
CD19+CD27+IgM- CD180 MMI	73.5 ± 19.0	38.2 ± 28.5	<0.0001
CD19+CXCR4hiCCR7hiCD21lo (t-SNE) ^‡^	0.1 ± 0.1%	6.1 ± 19.1%	0.0001
CD19+CD21loCD11c+ (t-SNE) ^‡^	2.3 ± 1.2%	4.1 ± 3.8%	0.09
CD3+			
CD3+CXCR3+^†^	9.6 ± 8.1%	2.6 ± 2.4%	0.0004
CD3+CXCR3-^†^	45.6 ± 9.8%	37.1 ± 13.4%	0.08
CD3+CD4+			
Total CD3+CD4+^†^	35.1 ± 6.6%	24.3 ± 7.8%	0.0001
CD3+CD4+CD45RO-CCR7+ (CD4+ Naive)^§^	27.7 ± 9.0%	31.8 ± 17.8%	0.89
CD3+CD4+CD45RO+CCR7+ (CD4+ Central Memory)^§^	18.5 ± 4.9%	15.7 ± 5.9%	0.23
CD3+CD4+CD45RO+CCR7- (CD4+ Effector Memory)^§^	13.5 ± 3.9%	11.3 ± 6.9%	0.13
CD3+CD4+CD45RO-CCR7- (CD4+ Effector)^§^	2.2 ± 1.2%	3.4 ± 3.0%	0.19
CD4+CD45RO-CCR4-CCR6-CXCR3- (Th0)^§^	27.9 ± 8.8%	31.9 ± 16.1%	0.64
CD4+CD45RO+CCR4-CCR6-CXCR3+ (Th1)^§^	2.8 ± 2.0%	0.8 ± 0.8%	0.0002
CD4+CD45RO+CCR4-CCR6+CXCR3+ (Th1Th17)^§^	1.1 ± 2.1%	0.1 ± 0.1%	0.01
CD4+CD45RO+CCR4+CCR6-CXCR3- (Th2)^§^	10.1 ± 4.6%	11.1 ± 4.7%	0.66
CD4+CD45RO+CCR4+CCR6-CXCR3+ (Th2CXCR3+)^§^	1.9 ± 1.3%	0.8 ± 0.6%	0.003
CD4+CD45RO+CCR4+CCR6+CXCR3- (Th17)^§^	1.5 ± 1.4%	1.3 ± 1.2%	0.57
CD4+CD45RO+CCR4+CCR6+CXCR3+ (Th17CXCR3+)^§^	0.6 ± 0.8%	0.1 ± 0.1%	0.03
CD3+CD4+CXCR5+PD1+ (T follicular helper) ^§^	0.4 ± 0.2%	0.1 ± 0.09%	0.0001
CD3+CD4+CCR4+CD25+CD127- (Treg) ^§^	2.2 ± 0.8	2.7 ± 2.0	0.88
CD3+CD4+CXCR4hiCD38- (Cluster “A”)^§^	0.3 ± 0.3%	5.5 ± 8.6	<0.0001
CD3+CD4+CD27-PD1+ (Cluster “B”)^§^	1.5 ± 1.6%	3.2 ± 3.3%	0.25
CD3+CD8+			
Total CD3+CD8+^†^	13.7 ± 4.3%	11.9 ± 8.1%	0.40
CD3+CD8+CXCR3+^§^	7.6 ± 5.6%	4.2 ± 4.6%	0.03
CD3+CD8+CXCR3-^§^	16.1 ± 6.9%	22.5 ± 11.5%	0.06
CD3+CD8+CD45RO-CCR7+ (CD8+ Naïve)^§^	5.5 ± 2.3%	4.5 ± 3.5%	0.26
CD3+CD8+CD45RO+CCR7+ (CD8+ Central Memory)^§^	3.3 ± 1.3%	1.8 ± 1.4%	0.002
CD3+CD8+CD45RO+CCR7- (CD8+ Effector Memory)^§^	8.9 ± 4.1%	8.8 ± 8.3%	0.42
CD3+CD8+CD45RO-CCR7- (CD8+ Effector)^§^	2.6 ± 2.7%	7.1 ± 6.8%	0.02
CD3-CD4-CD8-			
Total CD3+CD4-CD8-^†^	6.3 ± 4.7%	3.2 ± 2.7%	0.006
CD3-CD4-CD8-CXCR3+^§^	4.7 ± 6.5%	1.0 ± 0.7%	0.0002
CD3-CD4-CD8-CXCR3-^§^	5.7 ± 3.2%	6.0 ± 4.0%	0.71
CD19-CD3-			
CD3-CD19-CD14+ Classical Monocytes^†^	5.3 ± 2.1%	13.9 ± 9.1%	0.0003
CD3-CD19-CD14+CD16+ Non-classical monocytes^†^	6.2 ± 1.8%	8.6 ± 4.4%	0.10
CD3-CD19-CD14-CD16+ NK cells^†^	9.2 ± 4.1%	10.6 ± 6.3%	0.66
Biaxial “Cluster C” ^⁑^	2.3 ± 1.4%	6.3 ± 5.3%	0.0029
Biaxial “Cluster D” ^⁑^	1.9 ± 1.2%	3.9 ± 2.0%	0.0001

Values reported as average ± standard deviation. Statistical comparisons performed using Mann-Whitney U-test.

^†^Frequency of all PBMCs, ^‡^frequency of all CD19+ cells, ^§^frequency of all CD3+ cells, ^⁑^frequency of all CD3-CD19- cells.

### Autoimmune-Prone CD21lo and DN B Cell Subsets Are Increased in IIM Compared to Healthy Controls

**Figure 2:**
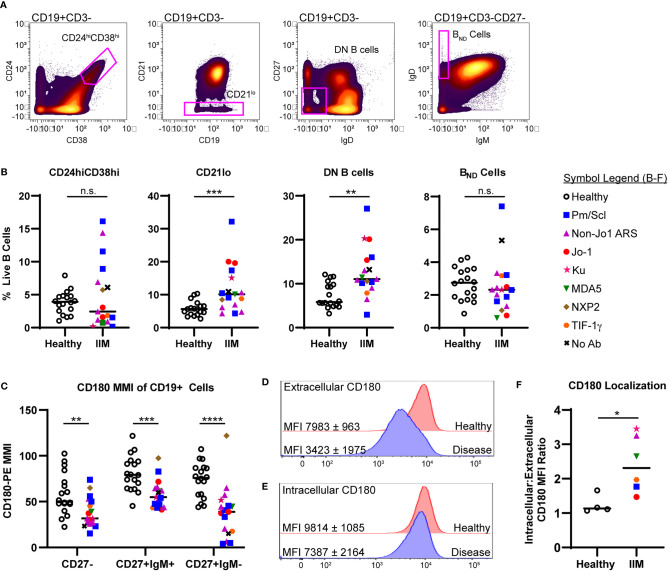
Autoreactive-prone CD21lo and DN B cells and CD180 surface expression are reduced in IIM patients. **(A)** Mass cytometry gating scheme to identify CD19+CD24hiCD38hi transitional B cells, CD19+CD21lo cells, CD19+CD27-IgD- (DN B) cells, and CD27-IgD+IgM- (BND) cells in healthy controls (n=18) and IIM patients (n=17). **(B)** Population frequencies for panel A subsets are shown for each donor; symbols represent clinical autoantibody status as in **Figure 1**. **(C)** CD180/RP105 mean mass intensity (MMI) for naive and memory CD19+ populations. **(D)** Fluorescent cytometry data quantifying CD180 expression on the cell surface and **(E)** intracellularly for four heathy controls and six IIM patients. Data are expressed as the mean ± standard deviation. **(F)** Ratio of intracellular to extracellular CD180 mean fluorescence intensity (MFI). Statistical comparisons were performed using Mann-Whitney U tests, *p < 0.05, **p < 0.01, ***p < 0.001, ****p < 0.0001, not significant (n.s.).

Next, we used manual biaxial gating to investigate the previously described autoreactive CD24hiCD38hi transitional B cell (40), CD21lo/negative B cell (36), CD27-IgD- DN B cell (50), and B_ND_ cell (35) subsets as shown in **Figure 2A**. There was no difference in the frequency of CD24hiCD38hi transitional B cells or B_ND_ cells in IIM patients compared to healthy controls. However, IIM patients had increased CD21lo/neg and DN B cells compared to healthy controls (**Figure 2B**). CD21lo/negative cells were previously shown to be autoreactive and/or anergic (36). The frequency of CD21lo/negative cells can be increased in systemic lupus erythematosus (51), rheumatoid arthritis (36), juvenile idiopathic arthritis (52), and systemic sclerosis (53, 54).

Kikuchi et al. reported an RP105/CD180 lo B cell population that was increased in dermatomyositis (37). Examination of CD180 median mass intensity (MMI) revealed decreased expression on naïve, class-switched memory, and non-class switched memory B cells in IIM patients compared to healthy donors (**Figure 2C**). There was no difference in the CD180 MMI on CD19+ cells of patients taking or not taking prednisone (41.7 ± 3.7% v. 54.2 ± 17.5%, p=0.14). To evaluate whether surface CD180 expression was decreased due to global protein downregulation, fluorescence cytometry was performed on IIM patients and healthy controls to quantify extracellular versus intracellular protein. Extracellular CD180 median fluorescent intensity was decreased in IIM patients compared to healthy controls, whereas intracellular CD180 expression was not different (**Figures 2D–F**). These data are consistent with surface downregulation, rather than reduced expression of CD180 in IIM B cells.

### Circulating Subsets of CXCR3+ T Cells Are Decreased in IIM

**Figure 3:**
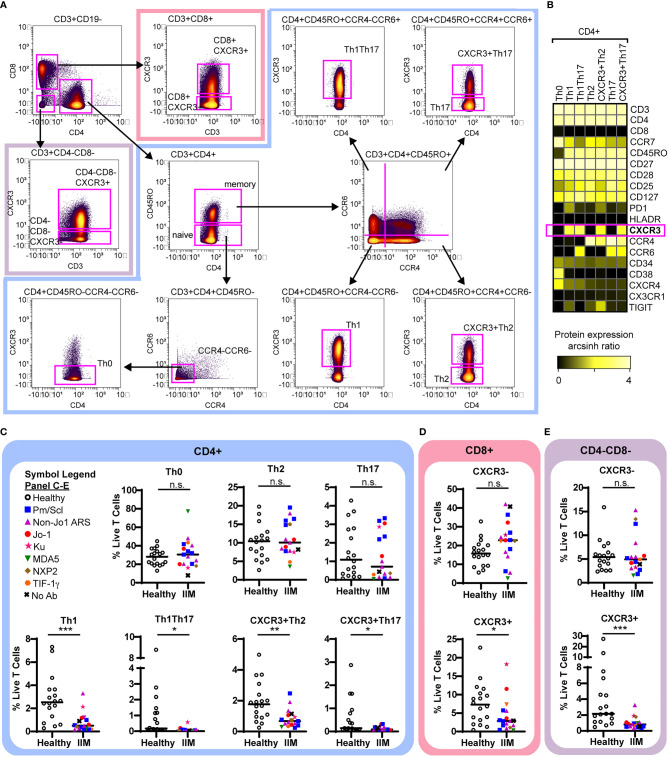
CXCR3+ T cell subsets are decreased in IIM. **(A)** Mass cytometry biaxial gating scheme to identify subsets of CD4-CD8-, CD4+, and CD8+ T cells for healthy controls (n=18) and IIM patients (n=17). **(B)** Expression heatmap of surface markers for CD4+ subsets displaying the arcsinh ratio by table’s minimum. **(C–E)** CXCR3- and CXCR3+ T cell populations were examined. Frequencies of biaxially gated T cell subsets are shown for each donor; clinical autoantibody status is indicated as in **Figure 1**; **(C)** CD4+, **(D)** CD8+, and **(E)** CD4-CD8- T cell populations. Statistical comparisons were performed using Mann-Whitney U tests. *p < 0.05, **p < 0.01, ***p < 0.001, not significant (n.s.).

We used previously defined biaxial gating schemes (34, 55) to evaluate CD4+ T cell subsets (**Figures 3A, B** and **Supplemental Figure S2A**) as defined in **Table 2**. There were no differences in CD4+ naïve, central memory, effector memory, or effector cells between IIM patients and healthy controls (**Supplemental Figure S2B**). IIM patients had an increased frequency of CD8+ effector cells but not CD8+ naïve, central memory or effector memory cells (**Supplemental Figure S2C**). However, as shown in **Figure 3C**, all CXCR3+ Th subsets, including Th1, Th1Th17, CXCR3+Th2, and CXCR3+Th17 cells, were strikingly decreased in IIM compared to healthy controls. There was no difference in the CXCR3- subsets Th0, Th2, and Th17. We additionally determined that the CXCR3+ subsets of CD8+ and CD4-CD8- T cells were decreased in IIM compared to healthy controls, but there was again no difference in the CXCR3- subsets (**Figures 3D, E**). There was no difference in the global frequency of CXCR3+ T cells in IIM patients taking prednisone compared to those not on prednisone (2.6 ± 3.2% v. 2.6 ± 1.7% of live PBMCs, p=0.37).

### Dimensionality Reduction Algorithms Reveal Two Abnormal CD19+ Cell Populations in IIM

**Figure 4:**
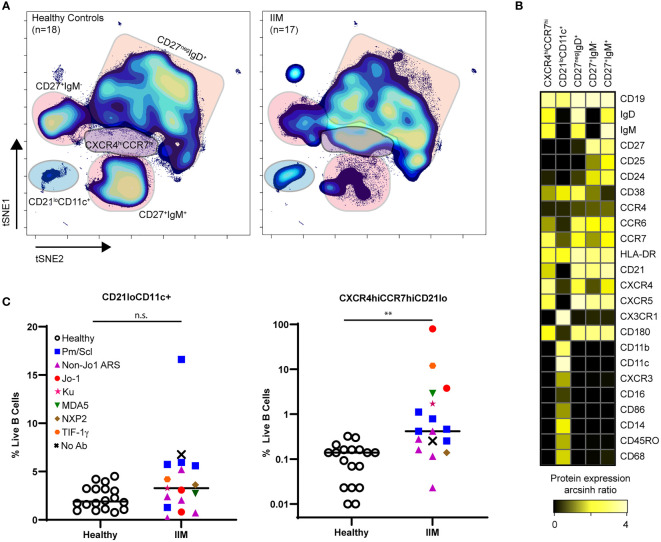
CD21loCD11c+ and CXCR4hiCCR7hi B cell subsets are increased amongst IIM patients. CD3-CD19+ B cells were visualized using t-SNE. **(A)** Concatenated t-SNE maps for all healthy controls (n=18) and IIM patients (n=17) showing traditional B cell subsets and two IIM-associated B cell subsets identified through manual gating of t-SNE plots. **(B)** Expression heatmaps of representative markers for CD21loCD11c+ and CXCR4hiCCR7hi subsets displaying arcsinh ratio by table’s minimum of channel median. **(C)** Frequencies of CD21loCD11c+ and CXCR4hiCCR7hi subsets are shown for each donor where various myositis associated and specific autoantibodies are coded as follows: Statistical comparisons were performed using Mann-Whitney U tests, **p < 0.01, not significant (n.s.).

Population identification using biaxial gating strategies relies on prior phenotypic marker knowledge. However, t-SNE plots represent multidimensional cellular information in 2D space, allowing for unbiased population identification and manual visual comparison (41). The t-SNE plots in **Figure 4A** and heatmap in **Figure 4B** show two atypical CD19+ cell subsets with varying frequencies in IIM patients as well as the previously seen decrease in class-switched and non-class switched memory CD19+ cell subsets (46) (**Figure 4C**). The CD19+CXCR4hiCCR7hi subset was increased in IIM patients compared to healthy controls. The second CD19+CD21loCD11c+ subset did not reach statistical significance but was increased in some IIM patients. The atypical populations identified *via* manual gating of t-SNE plots could also be found using biaxial gating (**Supplemental Figure 3**) and UMAP (**Supplemental Figure 4**). Thus, dimensionality reduction visualization of resulting data identified perturbed CD19+ cell subsets that were not captured by previous IIM studies.

### High Frequencies of Abnormal CD19+ Populations Predict the Presence of Altered Circulating CD4+ Populations

**Figure 5:**
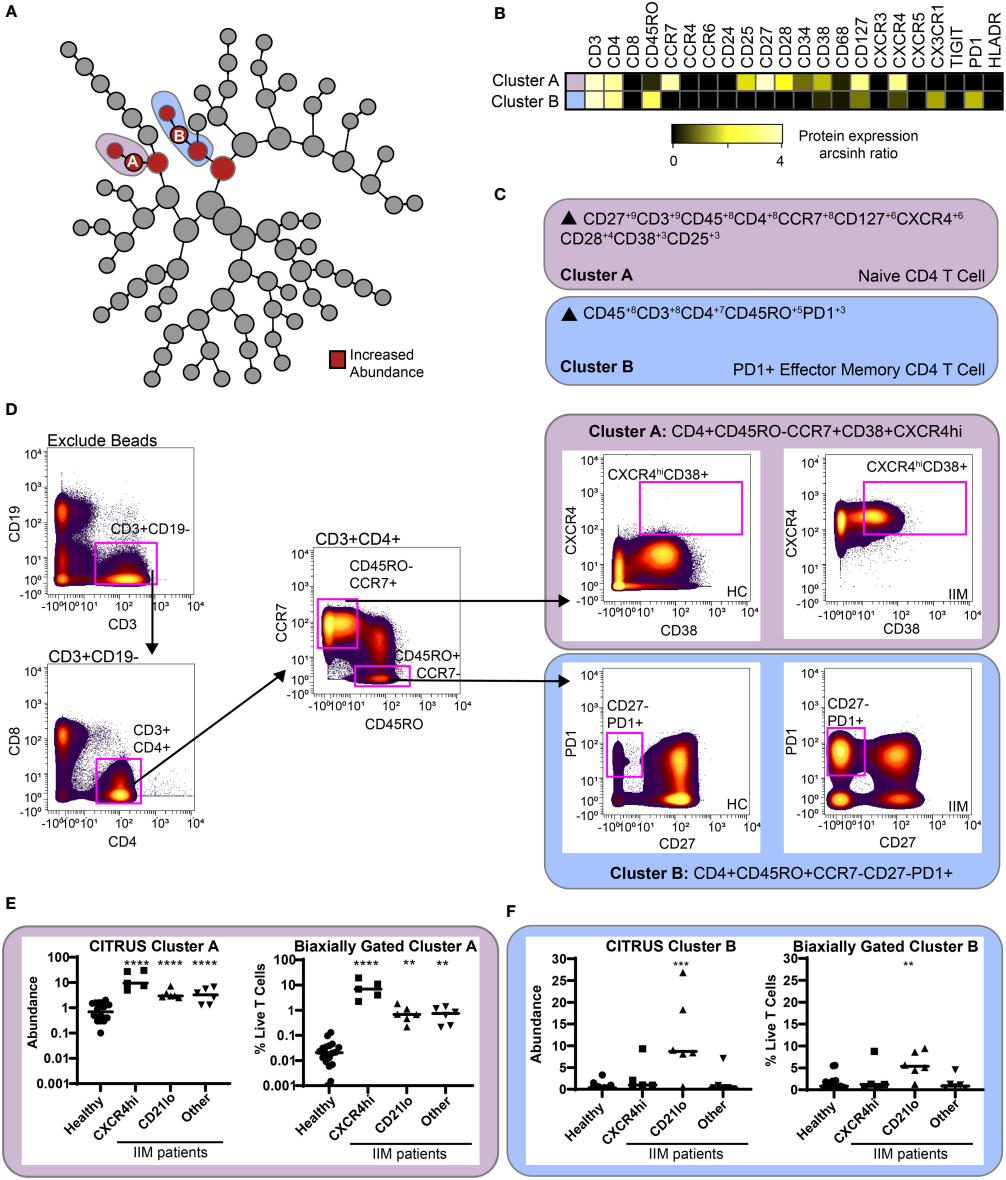
CITRUS identifies T cell clusters associated with abnormal CD19+CXCR4hiCCR7hi and CD19+CD21loCD11c+CD27- populations. CD3+ T cells were clustered *via* CITRUS using four groups (healthy control, n=18, CXCR4hi, n=5, CD21lo, n=6, and other B cell phenotype, n=6) and the nearest shrunken centroid algorithm. **(A)** Increased abundance of clusters A and B are predicted by the presence of the CXCR4hi and CD21lo subsets, respectively. **(B)** Expression heatmap of markers for Clusters A and B displaying arcsinh ratio by table’s minimum. **(C)** Algorithmic determination of biaxial gating scheme using marker enrichment modeling (MEM) for Clusters A and B Scale 1-10. **(D)** Biaxial gating scheme derived based on MEM definition; Cluster A: CD4+CD45RO-CCR7+CXCXCR4hiCD38+, and Cluster B: CD4+CR7-CD45RO+CD27-PD1+. Representative healthy control HC and IIM patient plots are shown. **(E, F)** CITRUS determined abundance and corresponding biaxial gating frequency for **(E)** Cluster A and **(F)** Cluster B are plotted for individual donors. If Kruskal-Wallis p<0.05, then *post-hoc* Mann-Whitney U tests were used to compare to healthy controls. **p < 0.01, ***p < 0.001, ****p < 0.0001.

CITRUS is a fully automated machine learning algorithm that identifies cell populations correlated with a particular biologic or clinical feature (42). CITRUS also incorporates regularized regression modeling to make statistically robust comparisons between groups, which was highly valued given the cohort heterogeneity. As specific B cell subsets can affect T cell phenotype (19, 20, 23), we used CITRUS to investigate correlations between CD19+CXCR4hiCCR7hi and CD19+CD21loCD11c+ cell subsets with T cell subsets. We created three mutually exclusive groups of IIM patients with high expression of CD19+CXCR4hiCCR7hi, CD19+CD21loCD11c+, or neither compared to healthy controls (**Table S7**). IIM patients assigned to the CD19+CXCR4hiCCR7hi group had ten-fold more cells in that population than the average for healthy controls. IIM patients assigned to CD19+CD21loCD11c+ group had two-fold more cells in that population than the average for healthy controls. As shown in **Figure 5A**, CITRUS identified increased abundance of CD4+CXCR4hiCD38hi naïve cells (Cluster A) in IIM patients defined by increased CD19+CXCR4hiCCR7hi cell frequency. CD4+CD27-PD1+ effector memory cells (Cluster B) were increased in the group with a high frequency of CD19+CD21loCD11c+ cells. To further characterize the phenotype of CD4+ cells in Clusters A and B, a heatmap of surface marker expression is shown for both populations. **Figure 5B** shows that Cluster A is comprised of a CD4+ naïve T cell subset with high levels of CCR7, CXCR4, and CD127 expression while Cluster B consists of a CD4+ effector memory cell subset positive for CX3CR1 and PD1. CITRUS also identified several populations of T cells that were decreased in IIM patients (**Supplemental Figure S5**).

To confirm this finding, marker enrichment modeling (MEM) computationally identified differentially expressed markers “enriched” in each cluster (45) to guide biaxial gating (**Figures 5C, D**). Biaxial population frequencies approximated the CITRUS-determined abundances (**Figures 5E, F** and **Table S8**). CD19+CXCR4hiCCR7hi and CD19+CD21loCD11c+ population frequencies were plotted against the biaxially gated frequency of the CD4+CXCR4hiCD38+ and CD4+CD27-PD1+ subsets, respectively (**Supplemental Figure S6**). We calculated Spearman’s correlation coefficients (**Table S9**) and confirmed a statistical correlation between CD19+CXCR4hiCCR7hi and CD4+CXCR4hiCD38+populations (r=0.62, p=0.009) and between CD19+CD21loCD11c+ and CD4+CD27-PD1+ populations (r=0.60, p=0.01). Subset frequencies for individual patients are shown in **Tables S10**. These data show that patients defined by specific CD19+ subset perturbations show correlative changes with particular CD4+ T cell subsets.

### An Increased Frequency of CD19+CXCR4hiCCR7hi Cells Predicts the Increase of Two Myeloid Populations

**Figure 6:**
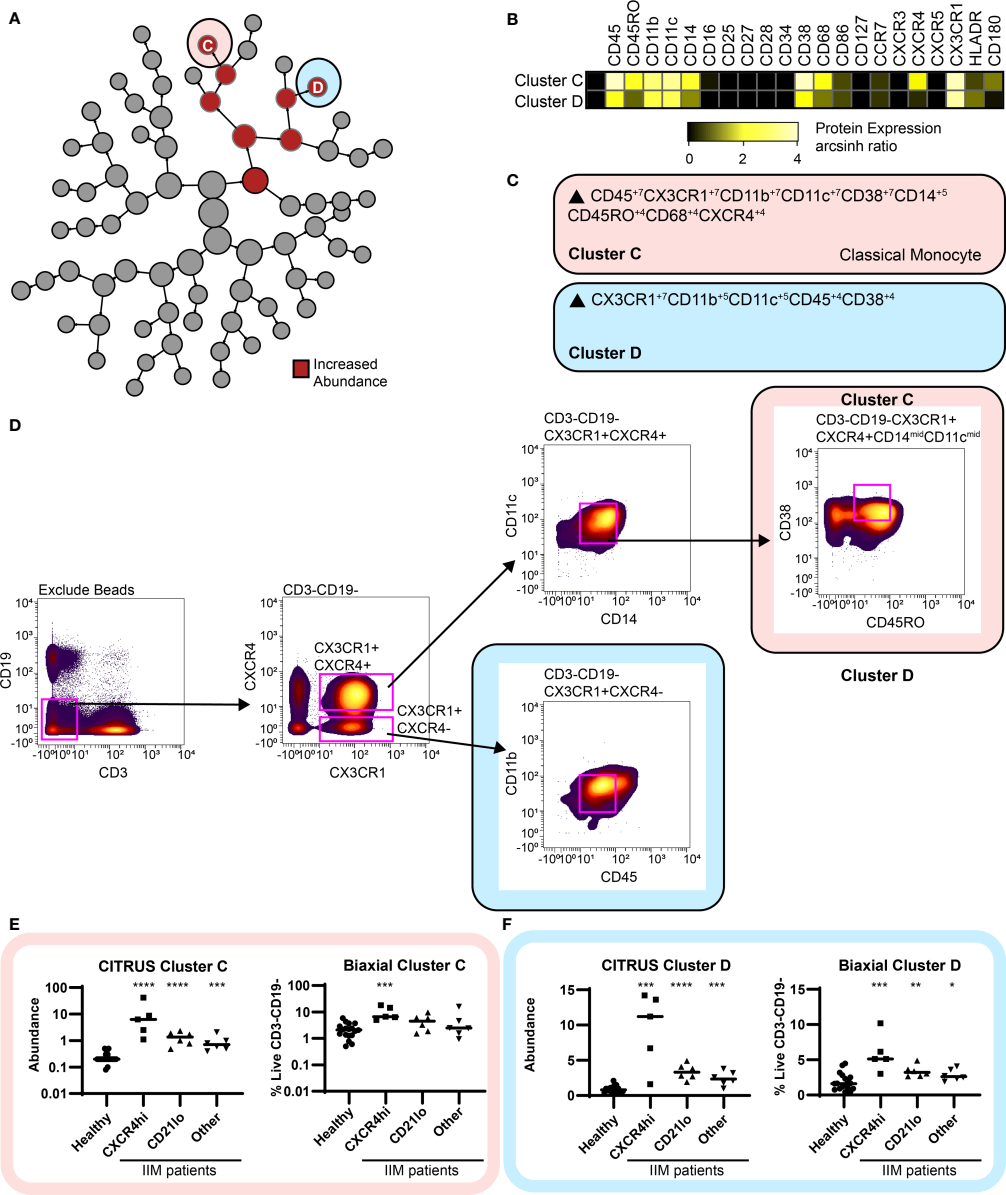
CITRUS identifies myeloid cell clusters that are increased in IIM. CD3-CD19- cells were clustered *via* CITRUS using four groups (healthy control, n=18, CXCR4hi, n=5, CD21lo, n=6, and other B cell phenotype, n=6) and the nearest shrunken centroid algorithm. **(A)** Increased abundance of Clusters C and D are increased across all IIM subsets. No clusters of decreased abundance were identified. **(B)** Expression heatmap of markers for Clusters C and D displaying arcsinh ratio by table’s minimum. **(C)** Algorithmic determination of biaxial gating scheme using marker enrichment modeling (MEM) for Clusters C and D. **(D)** Biaxial gating scheme derived based on MEM enrichment shown for a representative IIM patient. Additional t-SNE gating is shown in **Supplemental Figure 4**. **(E)** CITRUS determined abundance and corresponding biaxial gating frequency for Cluster C and **(F)** Cluster D. If Kruskal-Wallis p<0.05, then *post-hoc* Mann-Whitney U tests were used to compare to healthy controls. *p < 0.05, **p < 0.01, ***p < 0.001, ****p < 0.0001.

Using the previous IIM group assignments (**Table S7**), CITRUS clustered CD3-CD19- cells to correlate myeloid cell abundance with CD19+ cell phenotypes. Two clusters of increased abundance were predicted by having an increased frequency of CD19+CXCR4hiCCR7hi cells (**Figure 6A**). A heatmap of surface marker expression for both populations is shown in **Figure 6B**. While both Cluster C and Cluster D are classical monocytes, differential expression of CXCR4, CD45RO, and CD180 are readily apparent. MEM analysis confirmed Cluster C as a CD180+CXCR4+ classical monocyte and cluster C as a CD180-CXCR4- monocyte (**Figure 6C**). The MEM-derived biaxial gating scheme did not have clear visual cut-offs and final biaxial gating (**Figure 6D**) required the use of a training data set. Thus, myeloid cluster increases were further validated by t-SNE analysis (**Supplemental Figure S7**). Both the biaxial and t-SNE frequencies approximated the CITRUS abundance (**Figures 6E, F** and **Supplemental Figure S7D**). Spearman’s correlation coefficients confirmed a statistical correlation of the CD19+CXCR4hiCCR7hi subset with both the biaxially gated classical monocyte (r=0.61, p=0.01) and the CXCR4-CD180- myeloid population (r=0.66, p=0.01). The CD4+CXCR4hiCD38+ population also correlated to both the CD180+CXCR4+ (Spearman r = 0.64, p = 0.007) and CD180-CXCR4- (Spearman r = 0.64, p = 0.007) classical monocyte populations (**Supplemental Figure S5**). Subset frequencies for individual patients and group assignments are again shown in **Tables S10**.

## Discussion

Our key findings are summarized as follows. First, all IIM patients had decreased memory B cells, low RP105/CD180 surface B cell expression, and reduction of circulating CD3+CXCR3+ subsets, including Th1, Th1Th17, CXCR3+Th2, and CXCR3+Th17 cells. Second, we identified two distinct immune signatures, which we will term endotypes, amongst a cohort of IIM patients. Endotype 1 was characterized by increased CXCR4hi surface expression across all cellular compartments. Endotype 2 featured increased CD19+CD21loCD11c+ and CD4+CD27-PD1+ T cell populations. To our knowledge, these are the first immune endotypes to span multiple immune compartments in IIM.

Our B cell findings complement those of prior reports. Jo-1 autoantibody-positive IIM patients were previously reported to have decreased memory B cells (26), which we find across IIM patients independently of which myositis autoantibody was positive or which clinical disease manifestations were observed. Thus, our data suggest decreased peripheral blood memory B cells is a common feature of IIM that extends beyond Jo-1+ IIM patients. While the increase of CD24hiCD38hi (transitional) B cells did not reach statistical significance, some patients did have an increased frequency of this population, as previously observed in juvenile dermatomyositis (27). Decreased surface expression of RP105/CD180 was also found to be a common B cell feature in this clinically heterogeneous IIM patient cohort. CD180 is an alternative B cell signaling cascade capable of activating B cells either independently or synergistically with TLR9 or BCR engagement. CD180 is internalized after binding an unknown ligand, after which signaling occurs through an incompletely elucidated pathway (56). Thus, we postulate decreased surface expression of CD180 may reflect increased CD180 internalization and signaling in IIM patients.

We also identified a general decrease in circulating T cells expressing CXCR3. This may be due to CXCR3+ T cell migration into sites of inflammation. In support of this, interferon gamma and tumor necrosis factor alpha, which are increased in IIM, can induce secretion of the CXCR3 ligand CXCL10 by muscle fibers (57), and CXCR3+ T cells have been identified in the muscle biopsies of polymyositis, dermatomyositis, and inclusion body myositis patients (58). Of interest, CXCR3 ligands, CXCL9 and CXCL10, are increased in the serum of Jo1+ and SRP+ myositis patients (59), and CXCL10 is a validated disease activity biomarker for juvenile dermatomyositis (60).

The two immune endotypes identified here may have clinical correlations. Endotype 1 was hallmarked by increased CXCR4^hi^ surface expression across all immune subsets. Increased surface CXCR4 expression on circulating B and T cells was previously associated with increased disease severity in SLE (61) and 4/5 patients in our IIM cohort with this endotype were enrolled in the inpatient setting. We therefore postulate that the CXCR4^hi^ endotype is also associated with disease severity in IIM. Our sickest patient who was requiring extracorporeal membrane oxygenation also had the highest frequency of CXCR4^hi^ cells. Previously, CD4+CXCR4+ T cells were found to be correlated to high-resolution CT score, % predicted forced vital capacity, and 6-month mortality in IIM-ILD. *In vitro*, CD4+CXCR4+ T cells were found to stimulate fibroblast proliferation *via* IL-21, which was abrogated following co-culture with anti-IL21 blockade or tofacitinib (29). While CXCR4 can be upregulated by several stimuli (62–64), the cause of CXCR4 upregulation in IIM is currently unclear and represents a potential area of future research.

Endotype 2, comprised of the CD19+CD21loCD11c+ and CD4+CD27-PD1+ populations, may represent a pro-fibrotic phenotype. These CD19+ cells may represent DN2 B cells previously reported in SLE amongst patients with RNP antibodies, which are associated with skin and lung fibrosis. In those studies, DN2 B cells were characterized as CD19+CD21loCD27-IgD-CXCR5-CD24-CD11c+Tbet+, appeared to be plasma cell precursors, and were also exquisitely sensitive to TLR7 stimulation (65). The association of DN2 cells with RNP autoantibody positivity is of particular interest as this, like Pm/Scl, is an autoantibody that tends to be associated with features of systemic sclerosis, another fibrotic disease. In our study, most IIM patients with increased CD19+CD21loCD11c+ cells were Pm/Scl+. Alternatively, the numerous myeloid markers observed here raise the possibility that this CD19+ subset may not be of the B cell lineage (66, 67) or that the B cells may have acquired surface markers from follicular dendritic cells (68). CD4+PD1+ T cells have been reported in a number of fibrotic diseases including subglottic stenosis (69), idiopathic pulmonary fibrosis (IPF), and sarcoidosis (70). A trend towards increased frequency of CD4+PD1+ T cells has also been reported in systemic sclerosis (71).

Since these samples were obtained from an observational cohort, potential confounders, including steroid exposure, must be carefully considered. While sample size precluded formal multivariate and confounder analyses, qualitatively, there is no evidence that steroid exposure drove immune endotype. Four of the five patients with endotype 1 and 3/6 patients with endotype 2 were on at least 40 mg/day prednisone. Patients off all medications exhibited both immune endotypes. Additionally, CD180 surface expression and frequency of CXCR3+ T cells was decreased in all IIM patients, regardless of prednisone exposure. Future studies using larger IIM patient cohorts will be required to formally determine whether IIM medications contribute to the perturbed immune phenotypes identified here.

Studies identifying immunophenotypic signatures in rare diseases such as IIM are complicated by limited patient sample numbers, which limits the power to detect changes of modest magnitude or that are heterogeneously represented among the cohort. However, as our study demonstrates, supervised and unsupervised approaches successfully capture larger immune perturbations, which can provide important insight into disease biology. An additional limitation is the lack of concordant muscle or lung sampling precludes us from correlating our peripheral blood findings to end-organ inflammation and damage.

Overall, CyTOF facilitated simultaneous evaluation of multiple PBMC subsets using minimal sample with single cell resolution. Inclusion of hospitalized patients permitted us to investigate the entire spectrum of disease severity. Incorporating unsupervised analysis with traditional biaxial gating confirmed prior findings while also identifying novel subsets in a comprehensive and minimally biased way among IIM patients. The identification of two immune endotypes represents an important step forward in dissecting the immune phenotypes that contribute to clinical manifestations in IIM.

## MYSTIC Investigators

Ashley Blaske, Division of Rheumatology and Immunology, Vanderbilt University Medical Center, Nashville, TN, United States

Rosemarie B. Dudenhofer, Division of Allergy, Pulmonary, and Critical Care Medicine, Vanderbilt University Medical Center, Nashville TN, United States

Leslie J. Crofford, Division of Rheumatology and Immunology, Vanderbilt University Medical Center, Nashville, TN, United States^*^

Erin A. Gillaspie, Department of Thoracic Surgery, Vanderbilt University Medical Center, Nashville, TN, United States

Justin C. Hewlett, Division of Allergy, Pulmonary, and Critical Care Medicine, Vanderbilt University Medical Center, Nashville TN, United States

Joseph W. Houston, Division of Rheumatology and Immunology, Vanderbilt University Medical Center, Nashville, TN, United States

Susan Kroop, Division of Rheumatology and Immunology, Vanderbilt University Medical Center, Nashville, TN, United States

Kevin J. Myers, Division of Rheumatology and Immunology, Vanderbilt University Medical Center, Nashville, TN, United States

Stephanie G. Norfolk, Division of Allergy, Pulmonary, and Critical Care Medicine, Vanderbilt University Medical Center, Nashville TN, United States

Kristine Phillips, Division of Rheumatology and Immunology, Vanderbilt University Medical Center, Nashville, TN, United States

Todd W. Rice, Division of Allergy, Pulmonary, and Critical Care Medicine, Vanderbilt University Medical Center, Nashville TN, United States

Carla M. Sevin, Division of Allergy, Pulmonary, and Critical Care Medicine, Vanderbilt University Medical Center, Nashville, TN, United States

Bret Sohn, Division of Rheumatology and Immunology, Vanderbilt University Medical Center, Nashville, TN, United States

Melissa Warren, Division of Allergy, Pulmonary, and Critical Care Medicine, Vanderbilt University Medical Center, Nashville TN, United States

Erin M. Wilfong, Division of Rheumatology and Immunology, Vanderbilt University Medical Center, Nashville, TN, United States^*^

^*^Denotes members of the Writing Committee

## Data Availability Statement

The datasets presented in this study can be found in online repositories. The names of the repository/repositories and accession number(s) can be found below: http://flowrepository.org/id/FR-FCM-Z4MV.

## Ethics Statement

The studies involving human participants were reviewed and approved by the Vanderbilt University Medical Center IRB#141415. The patients/participants provided their written informed consent to participate in this study.

## Author Contributions

EW designed the study, performed patient enrolment, clinical chart abstractions, formal analysis of CyTOF data, data interpretation, and wrote the initial draft. TB assisted with CITRUS analysis, performed marker enrichment analysis, and aided in data interpretation. KV designed the CyTOF panel and performed data acquisition. CW performed data analysis and aided in figure creation. JI directed data analysis, interpretation of data, and substantially revised the manuscript. PK designed the study, aided in interpretation of data, and guided data analysis. LC designed the study, performed patient enrolment, directed data analysis and interpretation, and substantially revised the manuscript. RB directed data analysis and interpretation and substantially revised the manuscript. All authors have read and approved the final manuscript.

## Funding

This work was supported by CTSA award No. UL1TR000445 (EW, LC) from the National Center for Advancing Translational Sciences, the National Institutes of Health T32HL087738 (EW), KL2TR002245 (EW), K12HD043483 (RB), T32AR0590139 (KNV), K00-CA212447 (TB), R01CA226833 (JI), U54CA217450 (JI), P30CA68485 (VUMC Flow Cytometry Shared Resource), The Myositis Association (EW), Myositis UK (EW), Vanderbilt Faculty Research Scholars Award (EW), Vanderbilt Human Immunology Discovery Initiative, and the Porter Family Fund for Autoimmunity Research. The contents are solely the responsibility of the authors and do not necessarily represent official views of the National Center for Advancing Translational Sciences or the National Institutes of Health.

## Conflict of Interest

EW receives research funding from Boehringer-Ingelheim and is a member of their myositis ILD advisory board. JI was a co-founder and a board member of Cytobank Inc. and has engaged in sponsored research with Incyte Corp, Janssen, Pharmacyclics.

The remaining authors declare that the research was conducted in the absence of any commercial or financial relationships that could be construed as a potential conflict of interest.

## Publisher’s Note

All claims expressed in this article are solely those of the authors and do not necessarily represent those of their affiliated organizations, or those of the publisher, the editors and the reviewers. Any product that may be evaluated in this article, or claim that may be made by its manufacturer, is not guaranteed or endorsed by the publisher.

## Abbreviations

ARS, anti-tRNA synthetase syndrome; BCR, B cell receptor; CITRUS, cluster identification, characterization, and regression; ILD, interstitial lung disease; CyTOF, mass cytometry by time of flight; IIM, idiopathic inflammatory myopathies; MEM, marker enrichment modeling; MHCII, major histocompatibility complex class II; MMI, Median Mass Intensities; MFI, mean fluorescence intensity; PMBCs, peripheral blood mononuclear cells; t-SNE, t-distributed stochastic neighbor embedding.
